# Patients' experiences and perspectives regarding the use of digital technology to support exercise-based cardiac rehabilitation: a qualitative interview study

**DOI:** 10.3389/fspor.2024.1371652

**Published:** 2024-03-18

**Authors:** Anna Zeller, Johanna Gutenberg, Josef Niebauer, Rik Crutzen, Stefan Tino Kulnik

**Affiliations:** ^1^ Faculty of Medicine, Paracelsus Medical University, Salzburg, Austria; ^2^ Ludwig Boltzmann Institute for Digital Health and Prevention, Salzburg, Austria; ^3^ University Institute of Sports Medicine, Prevention and Rehabilitation and Research Institute of Molecular Sports Medicine and Rehabilitation, Paracelsus Medical University, Salzburg, Austria; ^4^ Department of Health Promotion, Care and Public Health Research Institute, Maastricht University, Maastricht, Netherlands

**Keywords:** artificial intelligence, cardiovascular disease, data protection, digital health literacy, mobile health, physical activity, secondary prevention, telemedicine

## Abstract

**Introduction:**

Despite the well-known benefits of exercise-based cardiac rehabilitation for the secondary prevention of cardiovascular disease, participation in cardiac rehabilitation programmes and adherence to secondary prevention recommendations remain limited. Digital technologies have the potential to address low participation and adherence but attempts at implementing digital health interventions in real-life clinical practice frequently encounter various barriers. Studies about patients' experiences and perspectives regarding the use of digital technology can assist developers, researchers and clinicians in addressing or pre-empting patient-related barriers. This study was therefore conducted to investigate the experiences and perspectives of cardiac rehabilitation patients in Austria with regard to using digital technology for physical activity and exercise.

**Methods:**

Twenty-five current and former cardiac rehabilitation patients (18 men and 7 women, age range 39 to 83) with various cardiac conditions were recruited from a clinical site in Salzburg, Austria. Semi-structured qualitative interviews were audio-recorded and transcribed verbatim. The analysis followed a descriptive phenomenological approach, applying the framework analysis method.

**Results:**

The sample was diverse, including interviewees who readily used digital devices to support their physical activity, exercise and health monitoring, and interviewees who did not. Simplicity, convenience and accessibility were highlighted as important facilitators for the use of digital technology, while annoyance with digital devices, concerns about becoming dependent on them, or simply a preference to not use digital technology were commonly stated reasons for non-use. Interviewees' views on data protection, data sharing and artificial intelligence revealed wide variations in individuals' prior knowledge and experience about these topics, and a need for greater accessibility and transparency of data protection regulation and data sharing arrangements.

**Discussion:**

These findings support the importance that is attributed to user-centred design methodologies in the conceptualisation and design of digital health interventions, and the imperative to develop solutions that are simple, accessible and that can be personalised according to the preferences and capabilities of the individual patient. Regarding data protection, data sharing and artificial intelligence, the findings indicate opportunity for information and education, as well as the need to offer patients transparency and accountability in order to build trust in digital technology and digital health interventions.

## 1 Introduction

The growing prevalence of cardiovascular disease (CVD) presents an increasing global challenge. Accounting for 18.6 million deaths per year in 2019, CVD remains the leading cause of death worldwide (1). Patients suffering from CVD and its sequelae such as myocardial infarction, heart failure and stroke face severe burden, including reduced quality of life, reduced exercise tolerance and a higher risk of hospital admissions and mortality (2). In Austria in 2019, the age-standardised CVD incidence was 654 per 100,000, the age-standardised mortality attributed to CVD was 151 per 100,000, and CVD accounted for 5,105 disability-adjusted life-years per 100,000 across all ages in both sexes (1). Prevention is of utmost importance to reduce morbidity and mortality caused by CVD (3).

Exercise-based cardiac rehabilitation is an evidence-based secondary prevention model that has been proven to prolong life and improve functional capacity, well-being and quality of life for individuals with CVD (4, 5). Exercise-based cardiac rehabilitation is a multidisciplinary intervention comprising clinical assessments, patient education, pharmacological therapy, exercise training, physical activity counselling, psychological support and support to address CVD risk factors and lifestyle modifications. In the setting of this study in Austria, cardiac rehabilitation provision is standardised according to national guidelines and organised in four phases: phase I refers to the acute hospital admission; phase II refers to a structured programme under medical supervision in an inpatient (3 to 4 weeks) or outpatient (up to 6 weeks) setting, with the main focus on improving physical performance; phase III refers to a 6 to 12 months outpatient programme, with the aim of supporting sustainability of lifestyle modifications; and phase IV refers to patients' independent lifelong secondary prevention by continuing the CVD preventive behaviour from the previous phases (6, 7). Notably, the structure and organisation of cardiac rehabilitation programmes can differ between countries (8).

Despite the well-known benefits of exercise-based cardiac rehabilitation, many patients who qualify for it based on their medical history do not participate in cardiac rehabilitation programmes, with reported participation rates among eligible CVD patients in Austria of 30% and 20% for phases II and III, respectively (9), and 30%–50% in other European countries (8). Moreover, there is limited adherence and carry-over from the well-supervised cardiac rehabilitation phases to patients' independent secondary prevention behaviour. The maintenance of regular heart-healthy physical activity and exercise, for example, constitutes a crucial secondary prevention behaviour, but the effectiveness of cardiac rehabilitation programmes for establishing long-tern physical activity habits is variable (10, 11).

Low participation in cardiac rehabilitation programmes and poor long-term adherence to CVD secondary prevention behaviours are important contributing factors for re-hospitalisation and high rates of morbidity and mortality. The underlying reasons for low participation and poor adherence are many and multi-faceted (12, 13), including patients' employment and family responsibilities, location of cardiac rehabilitation centres and resulting travel times for patients, lack of social support from family and friends, and lack of knowledge and low health literacy (i.e., an individual's ability to access, understand and act on health information; 14) of individuals with CVD.

Digital technologies have the potential to address or at least alleviate some of these reasons. The term “digital health” describes the implementation of digital technology in the context of healthcare, encompassing “electronic health” (i.e., the use of information and communications technology in the health domain), “mobile health” (i.e., the use of wireless technologies for the purpose of health), “telemedicine” (i.e., the provision of health services at a distance), and emerging areas such as the use of advanced computing sciences in big data and artificial intelligence (15). Numerous scientific publications describe the vast potential of digital health interventions to advance the care of individuals with CVD, for example by enabling home-based and technology-based cardiac rehabilitation across phases II, III and IV (16), by supporting regular physical activity and other heart-healthy lifestyle modifications through text-messaging programmes, smartphone applications and wearable devices (17), or by enhancing clinical decision-making through artificial intelligence-supported analysis of large volumes of patient data (18). Moreover, systematic reviews provide good evidence of patient safety of digital health interventions for cardiac rehabilitation, with some studies even reporting fewer associated adverse events in digital intervention groups than in control groups (19). However, attempts at implementing such digital health interventions in real-life clinical practice frequently encounter various barriers (20). From the perspective of CVD patients, two consistently reported barriers are poor digital literacy and skills (i.e., lack of understanding of, or lack of physical capabilities to interact with digital health interventions) and low acceptability (i.e., lack of perceived effectiveness and low use of digital health interventions; 20).

Insight into CVD patients’ experiences and perspectives regarding the use of digital technology can assist developers, researchers and clinicians in addressing or pre-empting these patient-related barriers. These patient perspectives can be incorporated in the design of digital health interventions and in the design of evaluation studies and implementation strategies for digital health interventions. But to date, there have not been any publications of such studies for the Austrian cardiac rehabilitation community.

The present study was therefore conducted to investigate the experiences and perspectives of cardiac rehabilitation patients in Austria with regard to using digital technology, in particular for physical activity and exercise. The study addressed the following research questions: What are the reasons for use or non-use of digital devices? What are obstacles to the implementation of digital health interventions? What is the user experience and acceptance of currently available digital technology? And what are patients' views on recent developments and challenges in digital health around data protection, data sharing and artificial intelligence?

## 2 Materials and methods

### 2.1 Study design

We conducted a qualitative study with semi-structured interviews to explore patients' experiences and perspectives regarding the use of digital technology to support exercise-based cardiac rehabilitation. The reporting of this study follows the Consolidated Criteria for Reporting Qualitative Research (COREQ; 21). The COREQ checklist is provided in Supplementary Appendix S1. In the overarching methodological orientation of this work, we took a phenomenological approach, aiming to explore the topic from the perspective of those who have lived experience of CVD and cardiac rehabilitation (22).

### 2.2 Setting and sampling

The study was conducted at the Ludwig Boltzmann Institute for Digital Health and Prevention in Salzburg, Austria. Participants were recruited from among current and previous cardiac rehabilitation patients at the University Institute of Sports Medicine, Prevention and Rehabilitation in Salzburg, Austria. The sampling strategy was purposive, aiming for diverse representation in terms of age, gender and time since cardiac event.

A target sample of 25 participants was possible within the study resources and timeline and expected to yield relevant breadth and depth of data. Eligible were adult patients with a diagnosed CVD who were current or previous participants in phase II cardiac rehabilitation and residents of the city of Salzburg or its surrounding areas. Excluded were individuals with limited German language proficiency. Forty-nine eligible patients were identified from patient records at the recruiting site and invited to take part in the study, either in writing by letter or in person by clinical staff at the site. Patients were provided with a study information leaflet including a contact for patients to inquire further about the study. Patients were given at least 48 h to consider their participation in the study. All patients who agreed to take part in the study gave written informed consent.

### 2.3 Data collection

The content of the semi-structured interviews was developed based on relevant literature and included questions to explore two major topic areas: physical activity in cardiac rehabilitation, and digital technology. The interview guide was piloted with members of the Ludwig Boltzmann Institute's service user advisory group, consisting of CVD patients who had attended cardiac rehabilitation. All interviews were conducted by JG either face-to-face in a private room at the Ludwig Boltzmann Institute or remotely via video call if preferred by the participant. Interviews lasted between 34 and 92 min. No other persons were present during the interviews. Each participant gave one interview. All interviews took place during July to October 2020. All interviews were conducted in German. Interviews were audio-recorded on two voice recorders. Opening questions were asked verbatim according to the interview guide, and follow-up questions were asked optionally and depending on the course of the conversation. An English translation of the interview guide is attached in Supplementary Appendix S2.

Concluding the interview, participants were asked to answer demographic and disease-related questions, and to self-complete the German version of the International Physical Activity Questionnaire Short Form (IPAQ-SF; 23) and the German TA-EG questionnaire, a measure of affinity for technology (24). The IPAQ-SF includes seven questions to capture the volume and intensity of physical activity during the past seven days, allowing an estimate of physical activity levels (low, moderate, high) based on metabolic equivalents of tasks. The TA-EG questionnaire comprises 19 statements about technology (enthusiasm in the interaction with technology, subjectively experienced competence, positive consequences and negative consequences of the usage of technology), and respondents rate the extent to which each statement applies to them. Ratings range from 1 to 5, with higher scores reflecting higher affinity for technology. Participants completed the questionnaires independently while the interviewer remained in the room, available to answer questions if needed.

### 2.4 Data analysis

For our data analysis, we applied a mixed deductive and data-driven inductive approach using framework analysis according to the steps described by Gale et al. (25): transcription, familiarisation with the interview, coding, developing a working analytical framework, applying the analytical framework, charting data into a framework matrix and interpreting the data.

All audio-recorded interviews were transcribed verbatim, partly by the interviewing researcher (JG) and partly by a professional transcription service. Transcripts were pseudonymised, removing any information that could identify the speaking participant. These transcripts, supplemented by the IPAQ-SF and TA-EG questionnaires, constituted the data for analysis.

Data analysis was conducted by AZ with methodological support from STK and using Delve qualitative analysis software (https://delvetool.com/). During the familiarisation process, AZ read and re-read the interview transcripts several times, listened to the interview recordings, and wrote down her thoughts and impressions in analytical notes. Due to the richness of the data, we decided to restrict the analysis to interview sections related to digital technology, specifically with a focus on understanding the facilitators and barriers to use of digital technology for physical activity and exercise. AZ first read several transcripts and conducted line-by-line coding, describing her interpretation of quotes. These codes gave rise to the initial framework development. As AZ coded further transcripts, the initial framework was discussed and reviewed iteratively with STK and RC until consent was achieved. Afterwards, the coding framework was applied to the remaining transcripts, including one open category (“other”) to allow inductive coding of passages that did not match any of the framework codes but were considered interesting and relevant regarding the aim of the study. The coding framework and definitions of codes are provided in Supplementary Appendix S3. Once all the transcripts were coded, the data was summarised by category and charted into a thematic matrix. The final step of the data analysis was the interpretation of the data by identifying characteristics and differences, generating typologies and integrating theoretical concepts (25).

### 2.5 Research team and reflexivity

At the time of the study, the female interviewer (JG) was a pre-doctoral researcher at the Ludwig Boltzmann Institute with a background in nursing, master's degree and previous experience in qualitative interviewing. Other than communicating and establishing rapport with participants who expressed interest in the study, JG did not have any prior relationship with study participants. Participants knew that the research was conducted by JG in her role as researcher at the institute. The female data analyst (AZ) was a medical student with a previously completed psychology degree and conducted the data analysis as a research project towards her medical degree. AZ worked with the transcripts only and had no contact with study participants. STK was a research group leader at the Ludwig Boltzmann Institute with a background in physiotherapy and a doctoral degree in clinical neuroscience. He led the study and provided supervision and methodological support to JG and AZ. RC was professor of behaviour change and technology. He provided methodological and content expertise in the study design and data analysis. JN was professor of cardiology, scientific director of the Ludwig Boltzmann Institute and medical director of the recruiting site. He provided operational support for study recruitment and content expertise in the study design and data analysis.

Our reflexive stance, and in particular the reflexive stance of AZ as the main data analyst, was to take a descriptive approach aligned with transcendental phenomenology. We sought to bracket our individual subjectivity and to remain vigilant to the bracketing work, so as not to bias the analysis and interpretation (22).

### 2.6 Rigour

We employed several strategies for enhancing scientific rigour in qualitative research. AZ recorded reflexive and analytic notes throughout the analysis and interpretation process and discussed these in regular peer review meetings with STK and RC. We did not conduct member checking of interview transcripts or analysis results with study participants. We used the questionnaire data (IPAQ-SF, TA-EG) to incorporate an element of triangulation to the qualitative analysis. In the reporting of this study, we followed an international reporting standard for qualitative research (21).

### 2.7 Ethical and regulatory considerations

This study was conducted according to standard ethical practice in healthcare research with humans (26). Study participation was voluntary, and all participants provided written informed consent. Participants were free to discontinue their participation without giving a reason and without incurring any negative consequences. The study was submitted for review to the medical research ethics committee of the county of Salzburg (reference number 1040/2020) and was exempt from formal ethical review due to its low risk.

## 3 Results

Eighteen (72%) male and seven (28%) female patients participated in the study. Participants' age ranged from 39 to 83 years, with a mean age of 65.1 (SD = 9.6) years for male participants and 69.4 (SD = 9.8) years for female participants. Fifteen participants (60%) reported high physical activity levels, two (8%) reported moderate physical activity levels and eight (32%) answered the IPAQ-SF questionnaire incompletely. The sample's affinity for technology according to the TA-EG questionnaire was slightly above average, scoring 2.9 (SD = 0.9) for “enthusiasm”, 3.2 (SD = 1.0) for “competence”, 3.6 (SD = 0.6) for “positive attitude” and 3.2 (SD = 0.8) for “negative attitude”. Participant characteristics are presented in Table 1.

**Table 1:** Participant characteristics.

Participant	Age group	Sex	Working	Cardiac history	Physical activity level	Affinity for technology^a^			
						Enthusiasm	Competence	Positive attitude	Negative attitude
P01	71–80	M	No	MI, heart surgery	High	3	3.3	3.6	3.2
P02	71–80	F	No	Dyspnea	High	2.4	2.8	3.2	3.2
P03	71–80	F	N/A	Heart valve disease	High	3.8	3.5	4	3
P04	61–70	M	Yes	Heart surgery	High	3.6	3.3	4	2.6
P05	71–80	M	No	MI, heart surgery	N/A	1.8	4.5	3.4	2.4
P06	71–80	F	N/A	Stent insertion	High	2.8	2	3.4	3.6
P07	71–80	M	No	MI, heart surgery	High	3.4	4	4.2	3.6
P08	61–70	M	No	MI	Moderate	3.6	4	3.8	3.2
P09	61–70	F	Yes	MI, heart surgery	High	3.4	2.5	4.6	3.2
P10	51–60	M	Yes	MI	High	3.8	4	3.6	3
P11	61–70	M	No	Cardiac arrhythmia	High	2.8	2	3	2.4
P12	31–40	M	No	Cardiac arrhythmia	N/A	N/A	N/A	N/A	N/A
P13	71–80	M	No	Stent insertion	High	3	4	3.6	3.4
P14	51–60	F	Yes	Cardiac arrhythmia	High	1.4	1.3	3.8	2.2
P15	51–60	M	No	Bypass surgery	N/A	3.2	4.3	3	1.4
P16	51–60	F	No	Diastolic insufficiency	High	1.2	1.5	2.6	3
P17	71–80	M	No	Heart surgery	High	5	4.5	5	3.4
P18	61–70	M	No	Stent insertion	Moderate	3.4	4.5	3.8	5
P19	41–50	M	Yes	Heart surgery	N/A	3.2	4	3.6	3.6
P20	81–90	M	No	MI	N/A	1.2	2.5	2.8	4.4
P21	51–60	M	Yes	Cardiac arrhythmia	N/A	2.6	2.5	3.8	4.2
P22	51–60	M	Yes	MI	N/A	2.2	2.3	2.8	2.2
P23	61–70	M	Yes	MI	High	2.4	2.5	4	3.2
P24	61–70	F	N/A	Angina pectoris	High	2.4	2.8	3.8	3.4
P25	61–70	M	No	N/A	N/A	3	4.3	4	3.4

F, female; M, male; N/A, not available/not answered.

^a^ Possible score range 1–5, higher score indicating higher affinity for technology.

**Figure 1:**
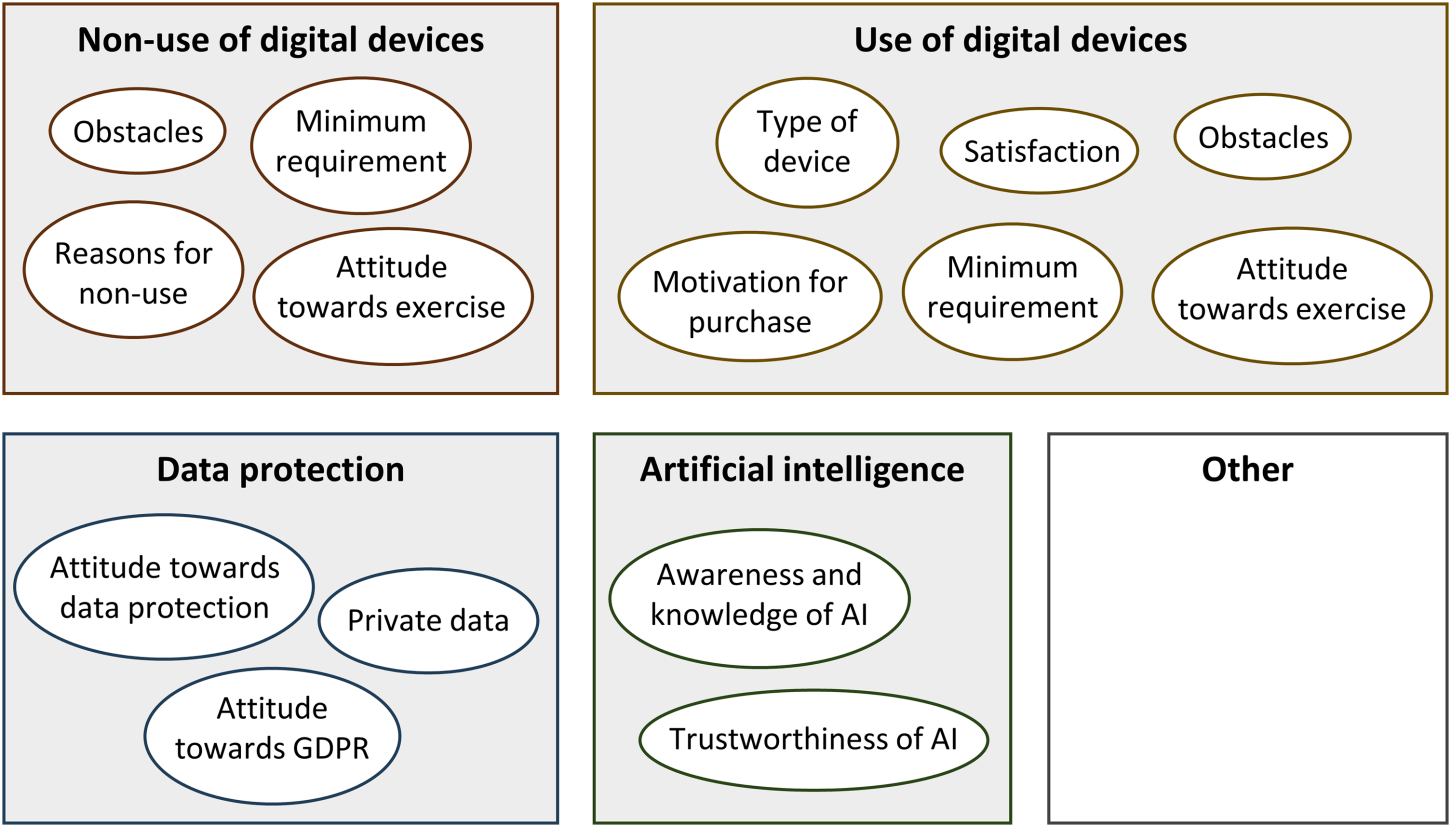
Overview of themes and codes. AI, artificial intelligence; GDPR, general data protection regulation.

Figure 1 gives an overview of themes and codes according to the coding framework (Supplementary Appendix S3). For this results section, complementary codes were combined and are presented together to offer meaningful descriptions under the following sub-headings: reasons for using digital devices; reasons for non-use of digital devices; type of technology being used; need for improvement and complaints; attitude towards data protection; preparedness to share personal data; General Data Protection Regulation (GDPR); communication of data protection regulations; awareness of artificial intelligence; and trustworthiness of artificial intelligence. The description of results is supported with direct quotes, which were translated from the original German to English by AZ and STK. Quotes include participant pseudonyms, allowing cross-referencing with participant characteristics in Table 1.

### 3.1 Reasons for using digital devices

Although all the participants in the study owned a smartphone, only a little over half used it for more than simply making phone calls. For analytic purposes, participants were categorised into users (*n* = 15, 60%) and non-users (*n* = 10, 40%) of digital devices, whereby those who reported using digital technology such as smartphone applications, smartwatches or other tracking devices to support a healthy lifestyle and exercise in the context of their cardiac rehabilitation were categorised as “users of digital technology”.

The majority of users explained that digital technology had been recommended to them during cardiac rehabilitation and that their awareness of the importance of regular exercise and health monitoring had been created by healthcare professionals:

*Since they have actually recommended that [the use of digital devices] in the cardiac rehabilitation centre. (P08)*

*[I use that] in order to move in a better way, in order to move more. That is quite important! (P03)*

For digital device users, features related to health monitoring were of great importance. The three most mentioned health monitoring aspects were heart rate, blood pressure and caloric consumption. Many participants were concerned about being able to track their heart rate during physical activity:

*Well, of course, I need a heart rate monitor on my watch, because the heart rate should not rise too much. (P01)*

*To me, the minimum requirement is the blood pressure measurement. (P04)*

*Well, the step counter is important to me, because it shows my calorie consumption, doesn't it? (P03)*

Some participants not only appreciated the ability to continuously monitor their health through digital devices, but also wished for extended monitoring capabilities, including longer battery life and the ability to monitor health during sleep:

*The battery life [needs to be improved]. Because, if you are out and about all day, it needs to work for more than a maximum of four hours, in order to monitor without interruption. (P04)*

Apart from being able to monitor health parameters, many participants enjoyed the motivating features of digital devices:

*It is simply nice; if I am out and about anywhere, the watch on my wrist is suddenly vibrating, and I realise that I have at least met today's minimum requirement. (P3)*

*It is kind of motivating! Right now, it is a little fun. (P08)*

Some interviewees also highlighted that use of digital devices provided them with a sense of security and encouraged them to reflect on their own health behaviour:

*I use that right now because it simply gives me a sense of security. (P23)*

*Well, it is kind of a reflection tool to me. (P08)*

One participant stated that their main reason for exercising and using digital devices to improve their overall health was grounded in self-love. The interviewee explained that any technology was useless, unless one took care of oneself and one's health and well-being:

*Actually, it is all about self-love. (P12)*

### 3.2 Reasons for non-use of digital devices

Reasons for not using digital devices in cardiac rehabilitation or for exercise were varied. While some patients claimed to have “too little time” for dealing with technologies, others expressed an aversion to digital devices, due to negative past experiences:

*I am annoyed by the sounds […] it stresses me out. (P16)*

*I once owned such a device. That means, it actually never really worked. (P25)*

Several participants stated that they actively avoided handling digital devices:

*I actually try to avoid such devices […] I am more the analogue type. (P14)*

*I am old-fashioned. (P15)*

Other participants outright rejected digital devices, finding them unnecessary:

*I think I don't need that. Because I got a kind of feeling. I can do things without that. I don't need that. (P20)*

Some also considered technology as a threat and expressed concerns about being under constant surveillance and controlled by technology:

*I don't want to be controlled by such a device! (P07)*

*That's my opinion. It is bad for people. (P16)*

The difference in attitudes between users and non-users of digital technology was also reflected in the TA-EG scores, which averaged 3.3 (SD = 0,7) vs. 2.3 (SD = 0,8) for “enthusiasm”, 3.4 (SD = 0.8) vs. 2.9 (SD = 1.2) for “competence”, 3.8 (SD = 0.5) vs. 3.4 (SD = 0.6) for “positive attitude” and 3.3 (SD = 0.7) vs. 3.0 (SD = 0.9) for “negative attitude”, respectively.

Notably, many participants among both users and non-users of digital technology mentioned their dependence on younger relatives when handling digital devices, describing that they relied on someone to regularly explain and show them new features and applications:

*My son got me a tablet for Christmas, and he showed me how to look at pictures. I cannot do much more than that with it, but I don't want to anyway. (P06)*

### 3.3 Type of technology being used

Participants stated that the devices they predominately used to monitor exercise and health parameters were smartphones and smartwatches. Most participants expressed their appreciation for simple handling and pre-installed health applications on their devices, such as the step counter function:

*Yes, the walking function is on it [the phone]. My 6,000 steps are on it. That has already been integrated on the smartphone, completely installed. (P02)*

*Yes, I have such an app. […] that means I have to always carry my phone with me. It exactly shows how many steps one takes or what one has done in a day. (P18)*

*[…] so, if I look at my watch today, I look at a quite good, new Suunto. That's where my step counter has been installed automatically. (P21)*

*Well, the Apple watch is quite convenient, […] insofar as I find all relevant applications in the main menu. (P10)*

Only a few participants reported using health and exercise-promotion programmes offered on television or online during the COVID-19 pandemic. Few participants also used more sophisticated devices such as global positioning system (GPS) sensors and other types of monitors incorporated into their bicycles or other sports equipment:

*Yes, that's the GPS on the mountain bike. It shows altitude and helps with orientation, geographically, kilometres, and distances and so on. […] That's actually my favourite device. (P04)*

### 3.4 Need for improvement and complaints

With regard to complaints about digital technology and suggestions for improvement, the most often mentioned suggestion for improvement was a call for more simplicity. Participants stated that they did not want to use devices if they were too complicated to handle. Additionally, it was highlighted that the screen design and the text format had to be accessible and appealing:

*It has so many features that I do not need at all. (P02)*

*Some devices go far too much into detail. (P13)*

*It is important that you don't have to search for applications again and again. It should be clearly visible. Otherwise, one loses one's temper. (P13)*

Some interviewees complained about the high level of dependence that technical devices would lead to:

*People rely too much on the feedback from those devices and not on things that your body is telling you. (P12)*

*I have already started to sometimes take the smartwatch off. […] to get a feel for it heart rate] again, otherwise, I would non-stop check the watch. (P23)*

A few participants criticised that the price of digital devices was too high and therefore unaffordable to them:

*Well, if that [smartwatches] would be available at a reasonable price, then I certainly would be interested. (P08)*

Some other users, however, stated that they were very pleased and satisfied with their devices and their handling.

*There is not really a thing that I would want to improve. I have not even thought about that. (P03)*

*There is absolutely nothing irritating about that [device]. (P02)*

### 3.5 Attitude towards data protection

When asked about data protection, interviewees displayed rather divergent attitudes. While some participants had a clear opinion on this issue, others stated that they did not care much about the topic of data protection, or that they simply found it to be an annoying issue:

*The problem is, if you want to use certain apps, you have to give your consent anyway. (P02)*

*[…] the whole data protection issue is the dumbest thing in the world that someone could possibly think of. (P21)*

Most participants stated that they were not too concerned about their data because they had “nothing to hide” anyway:

*To be honest, when it comes to data protection, I usually say: “I don’t have any secrets!” […] I don't take that too seriously. If they want to know something, let them know. (P02)*

*In principle, I go through life like that: if you are not really up to anything [secretive], nothing can happen to you with regard to data protection. (P12)*

In contrast, a few interviewees expressed their concerns about the use and transfer of data, as well as their wish for more transparency around data usage:

*That's why I say: I simply want to be in control of my data. (P10)*

*Well, when it comes to the internet, I am rather cautious. I am fully aware that all the data is certainly used somehow. In my opinion, more education and transparency on this topic would be quite important. (P16)*

### 3.6 Preparedness to share personal data

Some participants stated that they would be prepared to share all their personal data, and that they did not consider anything to be private data which should be handled confidentially:

*I don't have that kind of data that I would not be prepared to share. (P18)*

*I am like an open book. (P03)*

Most participants, however, reported at least one type of personal data that they were not willing to share. Many participants were most concerned about their financial data:

*When it comes to financial data, one must be particularly careful. (P04)*

*I have a small book back home. In there, all my bank data, the credit card number and stuff like that is handwritten and safe. I would never put that sort of data on my phone. (P17)*

To other participants, data about their leisure time, their current location or their family life was viewed as most important and confidential, and not to be shared:

*Well, I don't want to share my current location. I don't want to disclose any GPS data to any apps. (P15)*

*[I would not share] anything that has to do with my leisure time. (P12)*

Regarding the sharing of personal health data, interviewees' views were divided. Some participants stated that they would never willingly share their own health data, while others considered it their duty to share health data with healthcare institutions and providers, for example for health research purposes:

*Yes, of course [I would share my data] for research purposes! (P18)*

*Concerning medicine, I have to say, due to my personal medical history, I feel kind of obligated to return some favours to the health care system in any manner. (P19)*

Some of those who were not prepared to share their personal health data explained that they were afraid that this type of data could disadvantage them or be used against them by health and social care organisations:

*I don't have ELGA [Austrian electronic personal health record]. When it comes to health data, they pigeonhole people due to previous illnesses and stuff like that. (P24)*

*When it comes to health data, like serious diseases that are dealt with by the health insurance, I have to fear that it is passed on or traded. (P13)*

*If they are going to cut any benefits, then I have concerns! They have been talking about cutting certain things if one is obese. (P02)*

### 3.7 General data protection regulation

Most participants reported that they were not familiar with the meaning or the content of the European Union General Data Protection Regulation (GDPR):

*I have heard about it, but I don’t know what it is about. (P16)*

Other participants stated that they were well-aware of the GDPR and its content. Moreover, a few interviewees had personally made use of the regulation by asking companies and organisations to delete their data:

*[…] and that's when I told them, well, that I didn't [want] my data [to be used] any longer. Well, I think that worked automatically. (P18)*

*[…] so I sent a text message that referred to the GDPR and told them to delete all of my personal data and to never contact me again. The IT department even sent me a confirmation that everything has been removed. (P12)*

Personal attitudes towards the GDPR varied. While some participants expressed their appreciation for the regulation, the majority did not approve of it:

*I appreciate that. Yes, that is awesome! […] that I have the right to make use of it; this is very good! (P09)*

*No, this is totally unnecessary, because I know exactly whom I contact. (P24)*

*There is no point. (P05)*

*No, I have no need for that. […] I think I have nothing to hide, so I don't care at all. (P21)*

### 3.8 Communication of data protection regulations

When participants were asked about their opinion regarding the need to confirm data protection declaration or data privacy statements (for example, when downloading apps or programmes), most participants expressed their preference for a shorter, simpler and more appealing presentation of data protection regulations. Some suggested the use of keywords or the highlighting of essential passages:

*Of course, that could be designed in a better way. The key points could be highlighted, so there won't be misunderstandings. It could be reduced to the three or four most important questions. (P08)*

*That's the problem: who would ever have enough time to read and go through all that stuff? (P05)*

Others wished for more transparency, larger font size and a more accessible design:

*If the content is written in small print on the last page, no one is going to find that. (P21)*

*[…] if there was more transparency, that would be good! (P18)*

*[…] it is hard for the user to understand the content. […]no, it is too complicated. (P12)*

### 3.9 Awareness of artificial intelligence

With regard to awareness of artificial intelligence, the sample represented a range from those who had little or no awareness to those who had in-depth knowledge about artificial intelligence. Only very few participants stated that they had never heard of artificial intelligence before:

*No, [I have not heard about artificial intelligence yet]. What is it about? (P01)*

Most participants reported having some general idea about artificial intelligence and its areas of application:

*Well, I think artificial intelligence is becoming increasingly important in the medical sector. (P23)*

*Sure, Tesla, for example. Self-propelled cars, robots, drones. (P13)*

And some participants stated that they had personal experience with artificial intelligence in the past:

*Yes, I have been dealing with that. […] well, it is a double-edged sword. (P08)*

*You see, I have worked for an advertising agency, and artificial intelligence has been an important issue at work. […] everything that's happening in online stores or social media; in the background, there is always an algorithm and artificial intelligence and so on. (P19)*

### 3.10 Trustworthiness of artificial intelligence

In terms of the trustworthiness of artificial intelligence, participants' opinions diverged. Some participants explained that they would feel comfortable relying on artificial intelligence:

*I would rely blindly on. I would trust it. […] however, there is always somebody who initially had to program it, hence, the intelligence is still coming from people, in my opinion. (P17)*

*This is our future, for sure! Artificial intelligence, yes definitely! (P18)*

*Yes, of course [I would trust artificial intelligence]! It can be controlled. You can always recheck and do another test. (P02)*

Others were somewhat cautious and skeptical:

*All that, it is unrealistic to me! I don't comprehend it, that's very suspicious to me. Well, I mean, it is artificial. I have to say; I am always skeptical regarding anything artificial. (P03)*

*I don't think that artificial intelligence can or should be trusted blindly. (P19)*

*If I would receive a Tesla S with an autopilot as a gift, I would never hand the steering wheel to artificial intelligence or electronics. (P13)*

Some interviewees expressed concerns and fears over the use of artificial intelligence and its applications:

*Well, let me put it that way: if a machine suddenly is way more intelligent than its owner, then it's getting worrisome. Jobs are going to be lost! (P25)*

*Indeed, that's a bit creepy, to be honest! I am scared, yes! (P16)*

## 4 Discussion

We conducted semi-structured interviews to investigate the experiences and perspectives of 25 current and former cardiac rehabilitation patients in Austria with regard to using digital technology. The sample included individuals who readily used digital devices to support their physical activity, exercise and health monitoring, and individuals who did not. Simplicity, convenience and accessibility were highlighted as important facilitators for the use of digital technology, while annoyance with digital devices, concerns about becoming dependent on them, or simply a preference to not use digital technology were commonly stated reasons for non-use. Interviewees' views on data protection, data sharing and artificial intelligence revealed wide variations in individuals' prior knowledge and experience about these topics, and a need for greater accessibility and transparency of data protection regulation and data sharing arrangements.

### 4.1 Age-specific requirements

Although all participants reported owning a smartphone, only slightly more than half of the study group actually used smartphone applications and features beyond the simple function of calling. This observation is also mirrored in the Austrian general population of the corresponding age groups, although there has been an increasing tendency to use internet-based digital devices over the past 15 years (27). Reasons for non-use of digital technology may be attributed to generally low affinity for technology—as reflected in the low TA-EG scores for “enthusiasm” in our sample –, negative experiences related to technology, specific fears or concerns about using digital devices, or barriers related to older age (28–30). Krishnaswami and colleagues emphasise that age-specific barriers must be considered in the development and utilisation of technologies for older age groups (31). Participants in the present study suggested more simplicity of digital technology, including more appealing interfaces and less disrupting or irritating features, which echoes the findings of other studies. This poses the challenge to developers of digital health interventions to identify and be responsive to barriers across different age groups, and to design solutions that meet age-specific needs.

### 4.2 Dependence on support from others

Interviewees in the present study frequently reported a dependence on younger relatives, as many expressed a need for someone to explain features and applications to them, or to perform the installation and set-up of software and digital devices. Such lack of digital skills and limited knowledge and experience with digital applications present considerable obstacles to implementing digital health interventions in clinical practice (32). The active involvement of more digitally skilled spouses, partners, family members or friends in care processes might offer a solution (33, 34). Nevertheless, not all CVD patients can rely on such a support system, and healthcare providers should consider to also offer formal user training and support to accompany the implementation of a digital health intervention (31). In addition to equipping patients with the skills to use a specific digital health intervention, such formal training and support provides an opportunity to educate patients about the more general technological background, infrastructure and regulations, thereby helping to reduce reservations and concerns about digital health.

### 4.3 Peer support

The importance of strategies that promote peer support among cardiac rehabilitation patients is frequently reported in other studies (e.g., 35), but interestingly this was not a prominent topic in the present study. Digital technology offers many possibilities to create opportunities for peer support, by connecting patients to each other through remote communication technology (video calls, email, messaging) or social media platforms (Facebook, WhatsApp, etc.), or by incorporating specific behavioural techniques for peer support in digital health interventions, such as the sharing and affirming of one's behavioural intentions, goals and achievements in the digital peer group. In this context, previous studies have highlighted the importance of face-to-face contact and the value of developing a sense of community with other patients as well as with clinical staff (30, 33, 35, 36). While it is possible to achieve this through digital technology, for example using video conferencing platforms for face-to-face meetings and facilitating online communities via social media platforms, intervention developers may also consider blended formats including periodic in-person meetings for users of a digital health intervention.

### 4.4 Continuous monitoring and feedback

In Austria, cardiac rehabilitation programmes incorporate a focus on reducing the risk of recurrence by encouraging CVD patients to track their own health parameters (6, 37). Hence, it is not surprising that many interviewees in our study talked about their motivation and ambition to monitor heart rate, blood pressure and calorie consumption. Most users of digital technology perceived the monitoring functions of their digital devices as very beneficial for improving their physical activity and health. Some interviewees even expressed an interest in more extended and continuous monitoring capabilities of their devices, for example suggesting that devices should have longer lasting batteries to enable longer and continuous operation. Additionally, receiving frequent or continuous feedback seemed to not only provide users of digital technology with a sense of security, but to also serve as a motivating factor, as reaching one's goals was perceived as increasing one's motivation, self-confidence (self-efficacy) and self-esteem. Other qualitative studies of digital health interventions have reported similarly positive perceptions of self-monitoring and self-evaluation among CVD patients (e.g., 36). This was in stark contrast to interviewees in the “non-user” group, some of whom expressed that self-tracking and the generated data from it could lead to uncertainty, anxiety or fear. Some participants stated more nuanced concerns about relying too much on the feedback from digital devices and losing touch with feeling one's own body, while others decidedly considered technology and surveillance a threat. From an intervention developer's perspective this indicates a need to acknowledge these different sides of patients' experiences and to incorporate patients' views about useful features in the design of digital health interventions. Empowering users by giving them control over the extent and pervasiveness of digital monitoring functionalities may offer a solution for catering to different levels of engagement.

### 4.5 Data protection

Aside from the GDPR, which was introduced in 2018 and has significantly contributed to raising the profile of data protection issues across the European Union (38), the concept of “do-it-yourself data protection”—describing that comprehensive data protection needs to be done more and more by individuals themselves—has become increasingly relevant, as a growing number of actors are interested in individuals' personal data (39). This is compounded by the “digital divide”, i.e., the gap between those who have access to forms of modern information technology and those who do not (40, 41), placing individuals who lack knowledge, social status or resources to access digital technology or information at a disadvantage, also with regard to the protection of their personal data. Of note, the majority of interviewees in the present study appeared to care little about data protection. The statement “I have nothing to hide” was made frequently, expressing a sense of carefree light-heartedness that may reflect age, as it has been reported that older people are less likely to use tools or strategies that protect their personal data as compared to younger generations (39).

Conversely, some participants did express concerns about online security, called for more transparency and information about how their data was being processed, and stated a desire to be more in control of what was happening to/with their data and whether/how it was being shared, traded or otherwise used. However, this does not necessarily mean that these participants' online privacy behaviours were consistent with their attitudes, which can be explained by a knowledge gap (42). It has been reported that, although people express concerns about using and trading their personal data, they still share personal content online and accept that their data is taken and used by online providers. This observation indicates a lack of privacy literacy that prevents people from acting in a way that represents their beliefs and needs (42). Consistent with this observation, many participants in the present study stated that they provided their consent to privacy policies of websites and apps because, in their opinion, there was no alternative. Fostering knowledge about the technical aspects of online privacy and protection, laws and legal aspects, and strategies for individual privacy regulation is necessary in order to increase privacy literacy.

### 4.6 Sharing personal health data

There is now great international momentum towards enabling the sharing of personal health data to support healthcare delivery (“primary use of data”), and to facilitate access to health data for research and policy-making purposes (“secondary use of data”), for example through the establishment of the European Health Data Space ecosystem for data sharing (43). While some participants in the present study were fully prepared to share their personal health data with healthcare or research institutions, others expressed their preference to not share their personal data with anyone. Two main concerns were that health data could in some way be used against the person, and that there was a need for more transparency and information about data processing and trading. In particular, some participants expressed concerns that health insurances would deny payments based on information gained from personal health records. Public health research, however, relies on access to medical records and personal health data. If informed consent is required to access data from health records, this can lead to bias introduced through systematic differences between those who provide consent and those who do not. Therefore, it is essential to foster trust by offering patients information, education and transparency about data sharing processes and across all stages of data collection and processing (44). Public involvement in the discourse about the use of individual health records for healthcare research is of strategic importance to gain acceptance and reduce concerns and suspicion (45).

### 4.7 Artificial intelligence

Artificial intelligence has significantly advanced clinical care, for example by improving software for medical devices, facilitating the processing of large amounts of data and enabling greater precision of imaging technologies (46). In the past few years, the field has made great strides particularly in the development of large language models and natural language processing tools such as ChatGPT, with predictions that such applications of artificial intelligence could become valuable resources for clinical practice in the future (47). In this study, many participants viewed artificial intelligence with skepticism or even considered it worrisome or frightening. With regard to medical treatments, some participants stated that they would trust medical doctors more than they would trust any sort of artificial intelligence. This skeptical attitude might arise from a lack of information or a general objection to anything “artificial” as opposed to “natural”. Medical decision-making in general needs to account for uncertainty and often heterogeneous, erroneous, inaccurate or unknown data. While artificial intelligence might offer a way to integrate, fuse or map various data sources to support personalised decision-making and therapy prescription, it needs to be explainable and traceable to the extent that healthcare professionals have a possibility to understand how and why an artificial intelligence has arrived at a certain outcome (48). This level of transparency of the decision-making process is likely to also increase patients' trust. Some authors warn that the growth of artificial intelligence in healthcare might compound a trend from “hands-on” personal clinical practice to disembodied technological procedures (49). Others raise concerns whether artificial intelligence applications truly serve the patients' interests, or rather those who developed them (50). Against the background of these current academic discourses, the skeptical stance of many interviewees in the present study—albeit grounded mainly in intuitive reasoning rather than in an in-depth knowledge of the topic—certainly appears justified.

### 4.8 Technological solutionism

The terms “technological solutionism” and “technological fix” describe an ideology in which social or individual problems are considered discrete processes that can be improved and optimised by technological interventions (51). The steadily increasing body of evidence that demonstrates positive impacts of digital health interventions serves to amplify the widely publicised expectations that digital technology will (continue to) transform, or even revolutionise, healthcare (52, 53). This plays into a narrative of technological solutionism, placing emphasis on digital technology and its role for improving health. However, some authors criticise technological solutionism for denying the role of intrinsic and personal factors for individual health, as illustrated by one participant in the present study who was asked how CVD patients’ level of physical activity and exercise could be further increased and replied that “[…] actually, it is all about self-love!” (P12). There is therefore a balance to be struck, between, on the one hand, leveraging the fantastic capabilities of the many digital technologies that are available to us today and driving digital development forward, and, on the other hand, maintaining the focus on understanding individual patients' core needs and their personal motivating factors to incorporate these into personalised care that may include digital health interventions.

### 4.9 Limitations

We acknowledge several limitations to this study. Due to the recruitment of study participants via one clinical service, interviewees could have been influenced to some extent by clinical practice at the site, for example by rehabilitation professionals promoting digital technology as part of their practice. The sample size for this study was determined *a priori* by available resources, and we did not formally assess data saturation. However, acknowledging differences in interpretations and approaches to data saturation (54), we suggest that our sampling strategy achieved its purpose and resulted in a dataset that provided diverse views and depth of data. The analysing researcher (AZ) lacked the proximity to interviewees and interviews that comes with conducting interviews oneself, but she developed close familiarity to the data by repeatedly reading the transcripts and listening to the audio recordings of the interviews.

## 5 Conclusion

This qualitative interview study has provided insights into CVD patients' experiences and perspectives regarding the use of digital technology to support exercise-based cardiac rehabilitation. The findings support the importance that is attributed to user-centred design methodologies in the conceptualisation and design of digital health interventions, and the imperative to develop solutions that are simple, accessible and that can be personalised according to the personal preferences and capabilities of the individual patient. With regard to data protection, data sharing and artificial intelligence, the findings indicate opportunity for information and education, as well as the need to offer patients transparency and accountability in order to build trust in digital technologies and digital health interventions.

## Data Availability

The raw data supporting the conclusions of this article will be made available by the authors, without undue reservation.
