# Supplementary material for: Patients' experiences and perspectives regarding the use of digital technology to support exercise-based cardiac rehabilitation: a qualitative interview study
Source: Front Sports Act Living. 2024 Mar 18;6:1371652. doi: 10.3389/fspor.2024.1371652 (PMC10986307; doi:10.3389/fspor.2024.1371652)
Supplement: Supplementary file 1 [file Table1.pdf]

Supplementary Material

S1. Consolidated Criteria for Reporting Qualitative Research (COREQ) checklist. Pages 2-3

S2. Interview guide (translated from the original German). Pages 4-10

S3. Coding framework and associated definitions. Pages 11-12

S1. Consolidated Criteria for Reporting Qualitative Research (COREQ) checklist.

Topic	Item No.	Guide Questions/Description	Reported on Page No.
**Domain 1: Research team and reflexivity**			
Personal characteristics			
Interviewer/facilitator	1	Which author/s conducted the interview or focus group?	Methods
Credentials	2	What were the researcher's credentials? E.g. PhD, MD	Methods
Occupation	3	What was their occupation at the time of the study?	Methods
Gender	4	Was the researcher male or female?	Methods
Experience and training	5	What experience or training did the researcher have?	Methods
Relationship with participants			
Relationship established	6	Was a relationship established prior to study commencement?	Methods
Participant knowledge of the interviewer	7	What did the participants know about the researcher? e.g. personal goals, reasons for doing the research	Methods
Interviewer characteristics	8	What characteristics were reported about the inter viewer/facilitator? e.g. Bias, assumptions, reasons and interests in the research topic	Methods
**Domain 2: Study design**			
Theoretical framework			
Methodological orientation and Theory	9	What methodological orientation was stated to underpin the study? e.g. grounded theory, discourse analysis, ethnography, phenomenology, content analysis	Methods
Participant selection			
Sampling	10	How were participants selected? e.g. purposive, convenience, consecutive, snowball	Methods
Method of approach	11	How were participants approached? e.g. face-to-face, telephone, mail, email	Methods
Sample size	12	How many participants were in the study?	Methods
Non-participation	13	How many people refused to participate or dropped out? Reasons?	Methods
Setting			
Setting of data collection	14	Where was the data collected? e.g. home, clinic, workplace	Methods
Presence of non-participants	15	Was anyone else present besides the participants and researchers?	Methods
Description of sample	16	What are the important characteristics of the sample? e.g. demographic data, date	Methods, Ta[illegible]
Data collection			
Interview guide	17	Were questions, prompts, guides provided by the authors? Was it pilot tested?	Methods, A[illegible]
Repeat interviews	18	Were repeat inter views carried out? If yes, how many?	Methods
Audio/visual recording	19	Did the research use audio or visual recording to collect the data?	Methods
Field notes	20	Were field notes made during and/or after the inter view or focus group?	Methods
Duration	21	What was the duration of the inter views or focus group?	Methods
Data saturation	22	Was data saturation discussed?	Limitations
Transcripts returned	23	Were transcripts returned to participants for comment and/or	Methods

Topic	Item No.	Guide Questions/Description	Reported on Page No.
		correction?	
**Domain 3: analysis and findings**			
Data analysis			
Number of data coders	24	How many data coders coded the data?	Methods
Description of the coding tree	25	Did authors provide a description of the coding tree?	Appendix 3
Derivation of themes	26	Were themes identified in advance or derived from the data?	Methods
Software	27	What software, if applicable, was used to manage the data?	Methods
Participant checking	28	Did participants provide feedback on the findings?	Methods
Reporting			
Quotations presented	29	Were participant quotations presented to illustrate the themes/findings? Was each quotation identified? e.g. participant number	Results
Data and findings consistent	30	Was there consistency between the data presented and the findings?	Results
Clarity of major themes	31	Were major themes clearly presented in the findings?	Results, Figu
Clarity of minor themes	32	Is there a description of diverse cases or discussion of minor themes?	Results

From: Tong, A., Sainsbury, P., and Craig, J. (2007). Consolidated criteria for reporting qualitative research (COREQ): a 32-item checklist for interviews and focus groups. *Int J Qual Health Care* 19, 349–357. doi: 10.1093/intqhc/mzm042.

S2. Interview guide (translated to English from the original German).

Concept/Theme	Opening question(s)	Follow-up questions
Getting to know the interviewee	How are you?<br><br>Can you briefly talk about yourself?	 <br>Life situation, occupation, interests, hobbies, daily routines, family, friends
Physical activity	Now I would like to talk with you about the topic of physical activity. Can you talk about that?	What are your thoughts when I mention "physical activity"?<br>What does "physical activity" mean to you?<br>How does "physical activity" make you feel?<br><br>What other words come to your mind when I say "physical activity"?<br>What images come up in your mind when you think about "physical activity"?<br><br>What type of physical activity do you have experience with? *(e.g., sports, cardiac sports, training, exercise, physical education, physical exertion, hiking, running, jogging, cycling, etc.)*<br>How does ………. make you feel?
Physical activity recommendations in cardiac rehabilitation	What would you say is the recommended amount of physical activity for someone after a heart attack, heart surgery, etc.?	Can you remember what was recommended to you personally?<br><br>How did you receive these recommendations?<br>How did you find that?

		Do you have any suggestions, how these recommendations could be communicated better?
Behavioural objectives	What would you say are benefits of you meeting (or trying to meet) these physical activity recommendations?<br><br>What would you say are disadvantages of you meeting (or trying to meet) these physical activity recommendations?<br><br>Does anything else come to mind if you think about these recommendations for physical activity?	How do you benefit from physical activity?<br>What's in it for you?<br>What is the value of physical activity for you?<br><br>Any other comments of thoughts?
Normative referents	Thinking about these physical activity recommendations, can you think of any persons or groups of people who think that you should follow these recommendations – or that you should <u>not</u> follow these recommendations?<br><br>Please state which persons or groups of people would agree that you should follow these recommendations.<br><br>Please state which persons or groups of people would <u>dis</u>agree that you should follow these recommendations.<br><br>Sometimes we feel uncertain, and we make decisions based on the opinions of other people.	Who would give you moral support?<br>Who would encourage you?<br>Who would affirm you?<br><br>Who would <u>not</u> give you moral support?<br>Who would <u>not</u> encourage you?<br>Who would <u>not</u> affirm you?

	Can you think of any persons or groups of people who, like you, have a cardiac condition and who would probably follow these recommendations?	What kind of people are they?<br>What type of characters are they?<br>Can you think of anyone from your personal circle of friends or acquaintances?
	Can you think of any persons or groups of people who, like you, have a cardiac condition and who would probably not follow these recommendations?	What kind of people are they?<br>What type of characters are they?<br>Can you think of anyone from your personal circle of friends or acquaintances?
Control factors	Can you talk about which circumstances would enable or make it easier for you to follow these physical activity recommendations in the coming 3 months?	What would help you?<br>What would give you the necessary motivation?<br>Under which circumstances do you think that you will achieve it?<br>*If the person has recently been physically active:*<br>Think about the most recent occasion on which you made a conscious decision to do some physical activity: How did that come about?<br>What helped you do it?<br>What advice could you give to others?<br>What helps you to keep going?
	Can you talk about which circumstances would make it difficult or hinder you in following these physical activity recommendations in the coming 3 months?	What would hinder you?<br>Under which circumstances do you think that you will not achieve it?<br>*If the person had recently planned but then cancelled a physical activity:*<br>Think about the most recent time when you did not realise an opportunity for physical activity (e.g., cardiac sports, training session, etc.): How did that come about?<br>Why did it not happen?<br>How could this be changed?

		Can you think of any strategies how such situations could be avoided in the future?
Interventions for promoting physical activity	If you had the task to motivate people like yourself to be physically active, what would you suggest?	Have you got any ideas?<br>How would you go about it?
New technologies	What kind of "new" technologies do you currently use, or have you ever used?<br><br>We are thinking of so-called "digital" technologies and devices, such as smartphones, fitness trackers, etc.	How do you find these technologies and devices?<br>Do you have a favourite device or favourite technology?<br>What do you like about it?<br>What would you improve?<br>What do you dislike about it?<br><br>Have you ever used such devices/technologies to help you with your heart? (For example, when taking medication, to keep appointments, to help with changing your diet, or as a diary/notebook, etc.)<br><br>How could these devices/technologies help you personally with regular physical activity (cardiac sports, training, etc.)?<br><br>Can you think of any specific functionalities that would make regular physical activity more pleasant or more fun for you?<br><br>If you could make a wish for a technology for regular physical activity (cardiac sports, training, etc.), what should this technology be able to do?<br>What features should this technology have?<br>What would be the minimum requirements?<br>What would be disturbing? What would you avoid in any case?

Artificial intelligence	What comes to mind, when you think of the word "artificial intelligence”?<br><br>Artificial intelligence is defined as machines which can learn from experience, adapt to new incoming information and master tasks that require human-like thinking (e.g., chess-playing computers, self-driving cars).	Have you ever thought about artificial intelligence?<br><br>How would you rate your confidence in applications that use artificial intelligence?<br><br>Do you have experience with an application of artificial intelligence?
Data protection	In connection with these "new" technologies and devices, do you have any thoughts or experiences regarding data protection?<br><br>Some devices, apps or websites store data during use. Some of these data can be used to gain helpful information.<br><br>For example:<br><br>• Use of digital devices and services, e.g., websites and apps (e.g., Google search, search history and pages accessed / “browser history”)<br>• Activities and speed<br>• Location services (global positioning system)<br>• Daily routines, behavioural patterns<br>• Heart rate	Examples for devices: smart watches, fitness trackers, etc.<br><br>How would you see this, if this data were in your possession?<br>Or in the possession of the company that is hosting the app or the website?<br><br>What kind of data (information) would you be willing to make available?<br><br>What kind of data (information) would you not be willing to make available?

	How do you see the linking of this data with medical information?<br><br>For example:<br><br>• Heart rate<br>• Blood lipids<br>• Physical performance tests<br>• Medical diagnoses and prescriptions<br>• Weight, body mass index (fat/muscle percentage)<br>• Age<br>• Genetic and family information<br><br>How would you see it if, based on your non-sensitive data, a calculation or prediction of sensitive data is made? For example, if data from a fitness tracker about your physical fitness (non-sensitive data) is used to predict your life expectancy (sensitive data).<br><br>Such data could, for example, also contribute to scientific research: How would you see your data being used for this?<br><br>How can a good basis of trust be created when such data is made available for scientific research?<br><br>Have you heard of the General Data Protection Regulation (GDPR) that was	What is your understanding of this?<br>In your estimation, what consequences does to the GDPR bring for you personally?

	introduced across the European Union in 2018?<br><br>Have you ever submitted a request under the GDPR to have your personal data deleted or corrected?<br><br>When you use a smartphone app, do you usually read through the data privacy statement?	Do you have any suggestions, how a data privacy statement could ideally be designed?
Aim of the institute	The overarching aim of our research institute is to support people with cardiovascular disease in their regular physical activity.<br><br>We would be interested in your general opinion about this.	What is your view on this?<br>What would be important aims for you, with regard to our research?<br>What would make it valuable for you?<br>In your opinion, what would be important for us?
Other comments	Thank you for answering my questions.<br><br>If there is anything else you think is important, please let me know.	

S3. Coding framework and associated definitions.

Non-use of digital devices				Use of digital devices						Data protection			Artificial intelligence		Other
Obstacles	Minimum requirement	Reasons for non-use	Attitude towards exercise	Type of device	Satisfaction	Obstacles	Motivation for purchase	Minimum requirement	Attitude towards exercise	Attitude towards data protection	Private data	Attitude towards GDPR	Awareness and knowledge of artificial intelligence	Trustworthiness of artificial intelligence	
1	2	3	4	5	6	7	8	9	10	11	12	13	14	15	16

(1) & (7) *Obstacles.* Statements were labeled with this code in response to the prompt "What do you find annoying about digital technologies?" or if participants mentioned barriers or aversion to using digital devices.

(2) & (9) *Minimum requirement.* Statements were labeled with this code in response to the question "What are the minimum requirements that a device needs in order to be useful for you?" or if participants expressed preferences for digital devices.

(3) *Reasons for non-use.* Statements were labeled with this code if they included explanations regarding why participants did not use digital devices. This could include reasons related to personal characteristic features or daily life habits.

(4) & (10) *Attitude towards exercise.* Statements were labeled with this code in response to the question "What is your personal attitude towards 'exercise'?"

(5) *Type of device.* Statements were labeled with this code if they contained information about the type or the brand of devices that were used.

(6) *Satisfaction*. Statements were labeled with this code in response to the question "Are you satisfied with the usage of the device?" or if they related to information about participants' perception of devices and its handling, as well as suggestions for improvement.

(8) *Motivation for purchase.* Statements were labeled with this code if they explained participants' motives for acquiring digital devices or if they involved information about the way participants became aware of the possibility of using digital technologies.

(11) *Attitude towards data protection.* Statements were labeled with this code in response to the question "Have you ever encountered any experiences with data protection so far, in the context of 'new' digital technologies?" or if participants reported about their handling of data protection declarations in the usage of apps.

(12) *Private data.* Statements were labeled with this code in response to the question "Which data would you not be prepared to share?" or if participants explained what 'sensitive data' means to them.

(13) *Attitude towards GDPR.* Statements were labeled with this code in response to the question "Have you ever heard about the General Data Protection Regulation (GDPR), which was legislated by the European Union in 2018?"

(14) *Awareness and knowledge of artificial intelligence.* Statements were labeled with this code in response to the question "What comes to mind when you think of the word 'artificial intelligence'?"

(15) *Trustworthiness of artificial intelligence.* Statements were labeled with this code in response to the question "Would you trust artificial intelligence?"

(16) *Others.* Statements were labeled with this code if they did not match any of the codes listed above but were considered interesting and relevant regarding the aim of the study.
